# Impact of surface adhesion and sample heterogeneity on the multiscale mechanical characterisation of soft biomaterials

**DOI:** 10.1038/s41598-018-24671-x

**Published:** 2018-04-30

**Authors:** W. Megone, N. Roohpour, J. E. Gautrot

**Affiliations:** 1 Queen Mary University of London, Mile End Road, London, E1 4NS UK; 2 Institute of Bioengineering, Queen Mary, University of London, Mile End Road, London, E1 4NS UK; 3 Consumer Healthcare R&D, GlaxoSmithKline, St George’s Avenue, Weybridge, Surrey, KT13 ODE UK

## Abstract

The mechanical properties of soft materials used in the biomedical field play an important role on their performance. In the field of tissue engineering, it is known that cells sense the mechanical properties of their environment, however some materials, such as Sylard 184 PDMS (poly(dimethylsiloxane)), have failed to elicit such response. It was proposed that differences in the mechanical properties of such soft materials, at different scales, could account for these discrepancies. Indeed, the variation in the elastic moduli obtained for soft materials characterised at different scales can span several orders of magnitude. This called for a side-by-side comparison of the mechanical behaviour of soft materials at different scales. Here we use indentation, rheology and atomic force microscopy nanoidentation (using different tip geometries) to characterise the mechanical properties of PDMS, poly(acrylamide) (PAAm) and carboxymethyl cellulose (CMC) hydrogels at different length scales. Our results highlight the importance of surface adhesion and the resulting changes in contact area, and sample microstructural heterogeneity, in particular for the mechanical characterisation of ultra-soft substrates at the nano- to micro-scale.

## Introduction

The mechanics of soft materials for use in biomedical applications has been widely studied, such as for use in tissue engineering^1,2^, drug delivery^2,3^, wound healing^2,4,5^, and cell culture substrates for *in vitro* studies^6–8^. In particular, the mechanics of biomaterials has been shown to be very important in determining cell response and for implant design. Levental *et al*. showed that collagen crosslinking within the ECM and ECM stiffening was present within tumour growth and breast malignancy^9^. Furthermore studies have shown how, for cardiac repair, the heart valve leaflet stiffness is a key design feature, again showing the importance of the mechanics of soft materials used in bioengineering^10^.

**Figure 1:**
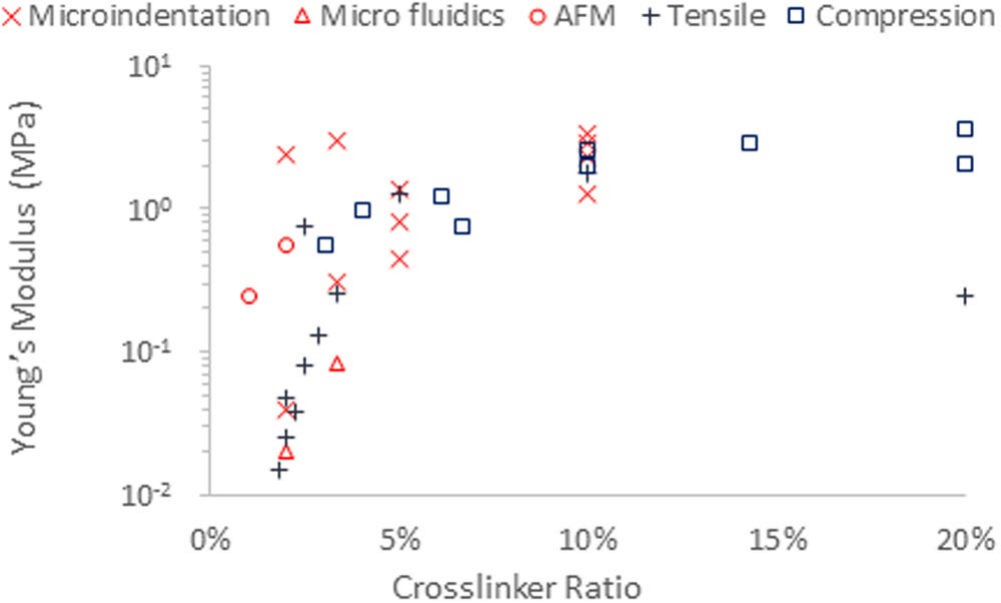
Young’s modulus of Sylgard 184 PDMS samples with varying base/crosslinker ratios highlighting the discrepancies between different testing methods, particularly at low levels of crosslinking (softer samples)^11–18^.

Despite the striking changes in cell phenotype observed on poly(acrylamide) (PAAm) gels with varying moduli (in the range of 1 kPa to 1 MPa), we previously reported the absence of apparent response of cells to Sylgard 184 poly(dimethyl siloxane) (PDMS) with moduli in the range of 0.1 kPa to 2 MPa^6^. However the origin of this apparent lack of response remains unclear. It was proposed that, at the local (nano- to micro-) scale and for small strains, the modulus of weakly crosslinked Sylgard 184 PDMS is significantly higher (up to two orders of magnitude) than what is reported for elastic and shear moduli (Fig. 1^11–18^). However, the origin of such behaviour (material stiffening at the nanoscale) is not clear and not supported by molecular modelling of polymer samples deformation^19–23^. Similar discrepancies between mechanical characterisation at different scales have been reported by others, although systematic studies of materials prepared in identical conditions and characterised at different length scales are still lacking. In addition, multiple scale mechanical characterisation of PDMS and PAAm has highlighted a very wide range of moduli reported for these materials, particularly for very soft samples (Fig. 1)^11–18^. However most mechanical testing techniques are optimised for traditional engineering materials which typically have high moduli (in the region of MPa – Gpa), making characterisation of soft biomaterials particularly difficult^24^. Hence, understanding the origin of such discrepancies and determining which techniques are most appropriate to use at different length scales, but also at different ranges of compliance, needs to be urgently addressed in order to tackle important questions in the field of biomaterials design and mechano-biology.

A number of hypotheses have been proposed to account for differences between bulk and local mechanics, such as heterogeneity within the material^25,26^ and adhesion between the sample and testing geometry^27,28^. Heterogeneity has been shown to lead to an increased modulus when using localised testing methods, such as nano indentation by AFM, however this is not reflected on the bulk mechanics^25^.

This phenomenon occurs when the local deformations applied for mechanical testing occur on the same length scale as the heterogeneity. In contrast, impact of sample heterogeneity on bulk mechanics will only be significant in cases where the filler (or hard nanomaterial/phase) forms a percolated network, forming a connective path between the geometries used for mechanical testing^29^.

Importantly, previous work has shown that the chemistry of the materials should be considered when determining a methodology for testing of a material at different length scales. Bush *et al*. introduced linear-harmonic interpolation of the Mooney-Rivlin and Boussinesq models, combined to compromise between nano and microindentation models allowing heterogeneity to be modelled using a finite, rather than small, strain model^25^. This method was designed to correct the contact model at larger relative indentation depths, however this does not take into consideration any wetting of the tip and associated adhesion phenomena. Sirghi and Rossi proposed an altered version of the Oliver Pharr method in order to take into account adhesion between the testing geometry and sample, however this method assumes a Hertzian contact which is only valid when wetting can be ignored^28^. Therefore, it appears important to investigate and quantify heterogeneity and adhesion simultaneously when characterising soft biomaterials at different length scales.

In this study, we investigate the mechanical properties of soft biomaterials (Sylgard 184 PDMS hydrophobic elastomers, hydrophilic PAAm and carboxymethylcellulose (CMC) composite hydrogels) and quantify discrepancies observed between moduli determined at nano- to macro-length scales, via rheology, indentation and atomic force microscopy. In particular, we quantify the combined impacts of heterogeneity of the corresponding materials and adhesion and wetting between the materials tested and the geometries of the instruments used for characterisation.

## Methods and Materials

### Materials

Toluene extra dry, 3-(trimethoxysilyl)propyl methacrylate, acrylamide, ammonium persulfate, NN′-methylbis(acrylamide), NNN′N′-tetramethylethylene diamine, hexamethyldisilazane, sylgard 184 PDMS, alyl bromide and PEG di thiol were all purchased from Sigma Aldrich. Aqualon sodium CMC was purchased from Ashland Chemicals.

### PDMS Sample Preparation

PDMS samples were prepared using the Sylgard 184 elastomer kit. Samples with 1 wt%, 2 wt%, 3 wt% and 10 wt% crosslinker were mixed thoroughly using a spatula before being put under vacuum to remove the bubbles. The samples were then cured at 60 °C for 3 h either *in situ* for use in rheology, on 13 mm glass coverslips for AFM indentation, and in 35 mm petri dishes for microindentation.

### PAAm Sample Preparation

Stock solutions were mixed according to the ratios shown in Table 1. The AAm stock solutions were degassed for a minimum of 30 min before use. Samples were then mixed with the crosslinking agents according to the following ratios 500 µL stock solution with 2.5 μL APS and 0.75 μL TEMED. Samples were then either cured *in situ* for use with rheology or between a 13 mm methacrylate functionalised glass cover slip and a hydrophobised plate for testing by AFM.

**Table 1:** Polyacrylamide stock solution compositions.

Gel	%AAm	%Bis	E (kPa)^1^	Ratio	40% AAm (mL)	2% Bis	UPW (mL)	Total vol. (mL)
*1*	5	0.02	2.0	1/250	0.625	50 µL	4.325	5
*2*	8	0.15	21.5	3/160	1	375 µL	3.625	5
*3*	20	0.375	115.0	3/160	2.5	937.5 µL	1.563	5
*4*	20	1.5	231.8	3/40	2.5	75 mg	2.5	5

### Allyl CMC Hydrogel Preparation

Sodium CMC was dissolved in a 1:1 mixture of DMF and DI water, the DMF was used in order to protonate the CMC, at a concentration of 1 mg/ml. Once the solution was homogenous Alyl Bromide was added at molar ratio of 30% alyl to CMC. The solution was heated to 70 °C and left overnight for the reaction to complete. The functionalised CMC was then precipitated in Acetone and dried. The final product was twice dissolved in DI water and precipitated in acetone to purify.

Functionalised CMC samples were then prepared by dissolving 100 mg/mL CMC in DI water and adding PEGDT and photoinitiator at the following alyl:PEGDT:PI ratio, 4:2:1. Samples tested by rheology were cured *in situ* and samples for AFM indentation were cured on 13 mm functionalised glass coverslips using UV light at 50 mW/cm^2^ for 2 min.

Samples containing silicon microbeads were prepared using the same method, however the microbeads were dispersed in the DI water before adding the functionalised CMC.

### Bulk Mechanical Characterisation by Oscillatory Rheology

Oscillatory rheology was used to characterise the mechanics of the series of PDMS samples using a TA Discovery HR-3 hybrid rheometer. The samples were cured *in situ* at 80 °C for 2 h and the gelation was monitored by an oscillating time sweep, with an oscillating frequency of 1 Hz and an oscillating displacement of 10^−4^ rad. Once cured the samples were then kept at room temperature for 5 min to cool and were then characterised using an oscillating frequency and stress sweep, and by transient stress relaxation. The frequency sweeps were performed from 0.1–100 Hz at an oscillating displacement of 10^−4^ rad and the stress sweeps were performed from 0.1–100 Pa at a frequency of 1 Hz. Finally stress relaxation was performed using a shear strain of 2% and a hold time of 300 s.

For testing of the PAAm samples the same method was used however the temperature was kept at room temperature and the curing time was a maximum of 1.5 h. The PAAm samples were also tested with and without the use of functionalising the rheometer geometry. In order to functionalise the rheometer geometry methacrylate functionalised glass cover slips were attached to the top and bottom geometry. The cover slips were held in place using Loctite Super Glue Ultra Liquid Control® with a tensile shear strength of 10 to 20 MPa (product specification for manufacturer). This ensures that the shear properties of the materials tested (with moduli below MPa) are not affected, but also enabled simple detachment of functionalised coverslip by solvent immersion (the glue is soluble in common organic solvents such as acetone). In order to minimise evaporation the rheometer geometry was enclosed in a solvent trap.

### Bulk Mechanical Characterisation by Microindentation Testing

Indentation testing was performed using an MTS coil driven universal test frame with a 500 N load cell. The samples were indented using a 3 mm conical flat ended punch. Indentation was done by stress relaxation testing with an indentation depth of 10% sample thickness at an indentation speed of 1 mm/s and a hold time of 300 s. The indentation data was then characterised using the Oliver Pharr method^30^.

### Local Mechanical Characterisation by Nanoindentation by AFM

Nanoindentation was performed using an Ntegra AFM rig. Bruker 8–10 contact cantilever tips were used with a nominal tip diameter of 15 nm. The cantilevers were calibrated by doing initial indentations on Silicon wafer. Samples were tested by doing a 10 × 10 grid of indentation at three different location on three different samples of each formulation respectively.

Wet AFM was performed with samples submerged in DI water, ethanol and PBS for the PAAm, PDMS and CMC samples respectively.

The indentation curves were then analysed using a custom made MATLAB script using the Oliver Pharr^30^, or Sirghi^28^ method depending upon whether adhesion was present.

## Results and Discussion

### Macro-to nanoscale mechanical properties of PDMS hydrophobic elastomers

**Figure 2:**
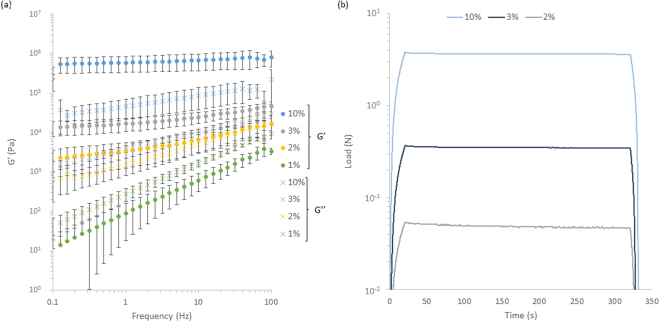
Bulk characterisation of PDMS by (**a**) Oscillatory rheology showing the frequency sweeps with frequencies from 0.1–100 Hz at an oscillating displacement of 10^−4^ rad for PDMS samples with % Crosslinker ranging from 1–10% a minimum of three repeats were conducted on each sample and (**b**) microindentation showing representative stress relaxation curves, of samples strained to 10% strain at a rate of 1%/s and a hold time of 5 min, each test was repeated three times. Error bars showing the standard deviation.

We characterised the bulk mechanical properties of PDMS samples crosslinked at different base to curing agent ratios via oscillatory rheology and microindentation first. The PDMS tested is a classical Sylgard 184 grade, commonly used in microfluidics, micro-contact printing and for cell culture^8,31–33^. It was crosslinked at different ratios, from 1 to 10 wt% (curing agent to base), in order to control its mechanical properties. Oscillatory rheology was carried out over the full range of compositions tested, but microindentation was not possible for the softest PDMS sample due to a lack of sensitivity of the load cell used. Results of bulk mechanical testing performed on PDMS are gathered in Fig. 2 and Supplementary Fig. S1.

Oscillatory rheology clearly indicated a strong increase in shear modulus with increasing crosslinker (Fig. 2a). This trend is also clearly observed in microindentation results (Fig. 2b). Frequency modulated oscillatory rheology also clearly evidenced the viscoelastic character of weakly crosslinked Sylgard 184 PDMS (1% crosslinker), which displayed a quasi-linear log-log relationship between shear modulus and frequency, with the modulus increasing by more than three orders of magnitude, from 1 Pa at 0.1 Hz to 3 kPa at 100 Hz. Such viscoelastic response was much weaker at higher crosslinker ratios and negligible at 10% crosslinker. Hence our data is in very good agreement with previously published micro-indentation data for similar Sylgard 184 PDMS materials^6^, confirming the bulk mechanical properties previously reported.

**Figure 3:**
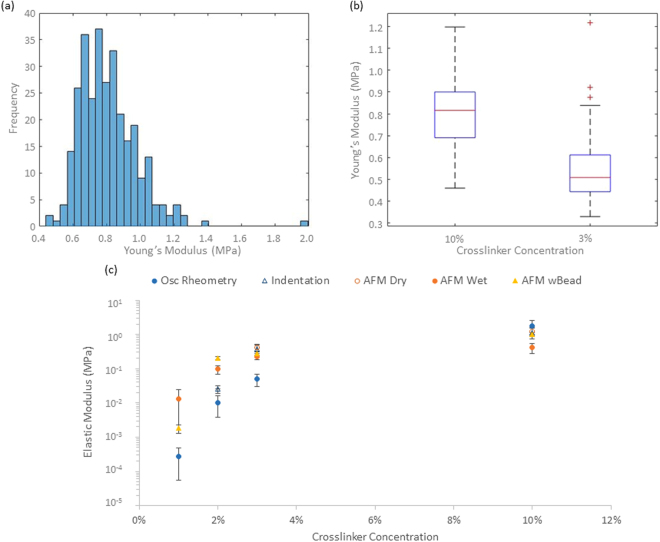
(**a**) Representative dry AFM histogram showing the spread in moduli value over three 5 × 5 μm locations with 100 indentations per location on a 10% crosslinker PDMS sample, (**b**) box and whisker plots showing the Young’s moduli obtained across the range of PDMS samples tested by dry AFM and (**c**) comparison of the bulk and local mechanical characterisation of the PDMS samples tested. All AFM experiments were conducted with an indentation depth of between 500 and 1000 nm and each curve was done over 1 s. AFM experiments were conducted on three different samples for each formulation and each sample tested in three different location with 100 indentations done on a 5 by 5 μm square.

**Figure 4:**
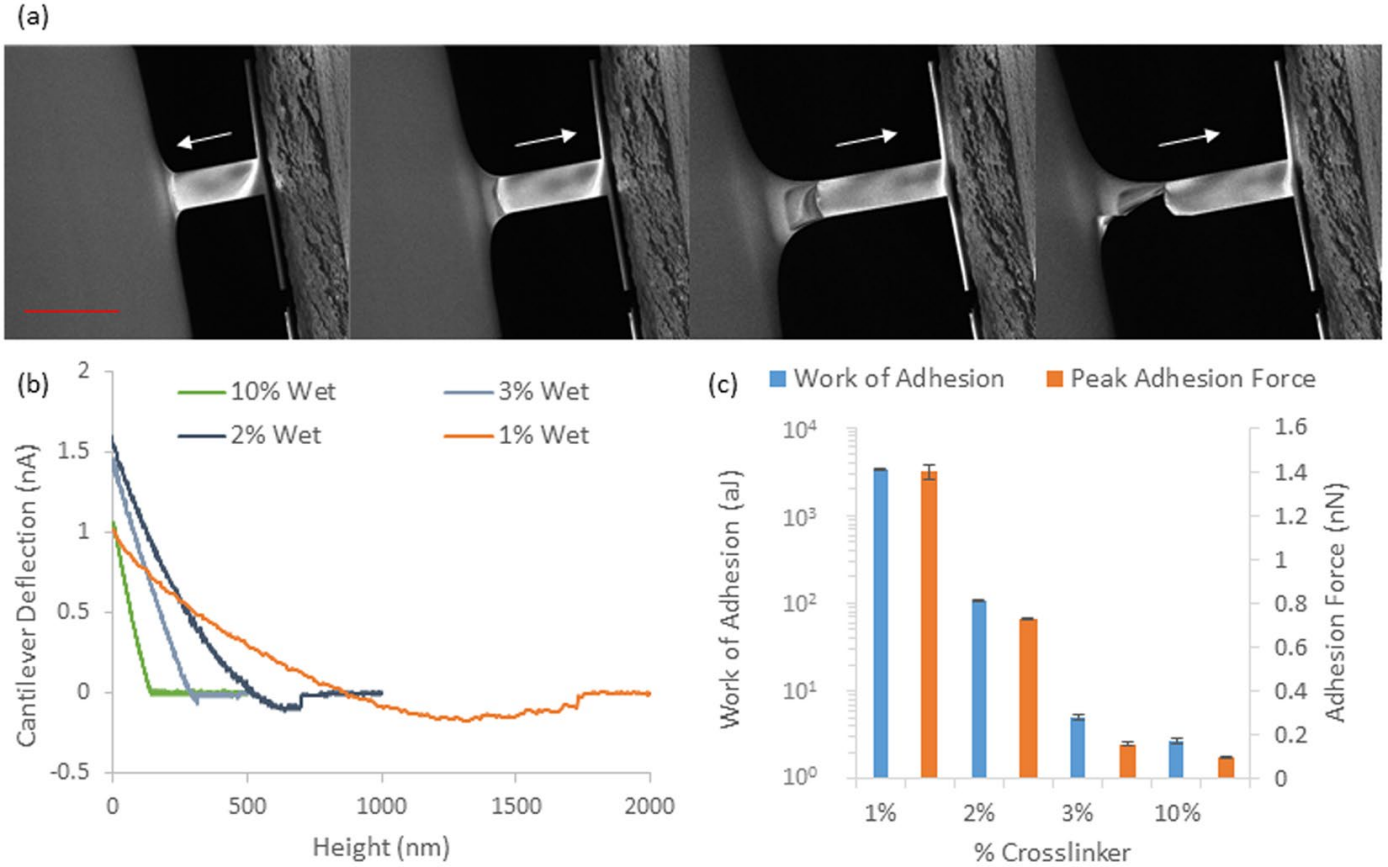
(**a**) SEM images of AFM tip interaction with 2% PDMS highlighting the adhesion between PDMS and Silicon Nitride AFM Tip snapping into and coming out of contact as indicated by the arrow. Scale bar is 100 um and (**b**) Nanoindentation by AFM retraction (lift) curves for PDMS samples tested wet in ethanol solution. All AFM experiments were conducted with an indentation depth of between 500 and 1000 nm and each curve was done over 1 s, each sample was tested in three different locations and 3 samples tested per formulation giving 900 data points per formulation (**c**) the average adhesion strength and peak adhesive force obtained by wet AFM.

We next examined the nanoscale mechanical properties of PDMS via nanoindentation (Figs 3a,b and S2). Whilst we measured elastic moduli in good agreement with bulk and shear moduli for the two stiffest PDMS formulations (elastic moduli of 0.41 MPa and 1.27 MPa for 3 and 10% crosslinker PDMS formulations, respectively), strong adhesion prevented tip detachment for the softest two samples (1 and 2%). This suggested important fouling of the cantilever surface by the soft PDMS formulation, a process that was clearly evidenced by *in situ* AFM-SEM imaging (Fig. 4a), hence, it is clear that Hertzian approximations cannot apply to weakly crosslinked Sylgard 184 PDMS materials. In order to reduce adhesion to cantilevers, AFM experiments were performed in ethanol solutions, which was shown to reduce adhesion of silicone materials^34^. In these conditions, the adhesion component was reduced significantly, including for the softest PDMS materials tested and tip detachment occurred within the working distance of our instrument (Fig. 4b). These AFM results were again in good agreement with bulk measurements for the PDMS samples tested with the highest moduli, but displayed significantly higher moduli, for softer PDMS (in particular at 1% crosslinker, which displayed a local modulus of 13 kPa compared to the shear modulus of 270 Pa measured via rheology) as shown in Fig. 3c. We noted relatively little heterogeneity in the samples characterized by AFM indentation both in dry and wet conditions (Figures S2 and S3), therefore suggesting that local heterogeneities are unlikely to account for the discrepancies observed (orders of magnitude) when materials were characterised at different lengths scales. Figure 4b indicates that there is still significant adhesion between the sample and indenter tip for the softest PDMS samples during nanoindentation even in wet conditions. The work of adhesion and maximum adhesive force were then characterised (Figs 4c and S4) to confirm that the level of adhesion between the sample and testing geometry was significantly higher for the softest PDMS sample (3,400 aJ for 1% crosslinker compared to 2.7 aJ for 10% crosslinker). Hence, the increase in the adhesion between the sample and AFM tip could explain the higher modulus obtained as the adhesion would change the wetting of the tip and hence the contact area between the sample and testing geometry. Overall, our data indicates a marked increase in adhesion observed as a function of decreasing Young’s moduli in colloidal AFM indentation (Figure S4). Hence, even in wet conditions, adhesion and wetting of soft PDMS substrates is causing significant deviation between local and bulk moduli.

In order to correct the wetting contribution observed in PDMS materials, we proposed to take into account the contact area between the probe and substrate, by performing adhesion tack tests on the softest PDMS sample (1%) using a 30 mm diameter probe with a plasma treated glass cover slip as the contact surface (mimicking the surface of the cantilever). The work of adhesion from the AFM tests was then calculated as the negative area under the force-displacement curve (i.e. the attractive force multiplied by distance)and the contact area assumed based on the following formula,

1 $$\frac{{A}_{i}}{{W}_{ad,i}}=\frac{{A}_{AFM}}{{W}_{ad,AFM}}$$

where A_i_ and A_AFM_ are the contact area for the 30 mm indenter probe and AFM tip respectively, and W_ad,i_ and W_ad,AFM_ are the work of adhesion for the indenter probe and AFM nanoindentation curves respectively. However this yielded contact areas for the AFM that were similar to that calculated using the assumptions present in the Oliver & Pharr method and the Sirghi method respectively. As a result nanoindentation by AFM was repeated across all the samples in ethanol solution however with a 4 μm colloidal bead attached as shown in Supplementary Fig. S2. Here the results followed a similar trend as observed with the previous AFM experiments, however for the 1% PDMS the modulus was much closer to that obtained by oscillatory rheology (1.8 kPa for colloidal AFM, compared to 13.2 kPa and 0.27 kPa for pyramidal tip AFM and oscillatory rheology, respectively).

The analysis of colloidal AFM indentation data was carried out using the Johnson^35^ Force-Displacement relation, which best describes a colloidal indenter within the linear elastic region^36^. However due to the adhesion present for the 1% PDMS the Sirghi method was adapted using the Johnson model with a colloidal indenter, rather than the Oliver Pharr, giving the following force displacement relation,

2 $$P=a(h-{h}_{f}{)}^{3/2}+b(h-{h}_{f})$$

where P is force, h is displacement h_f_ is the point at which the lift curve crosses the x axis and b is a constant. The Young’s modulus, E, is a function of the parameter a in equation 2, as follows:

3 $$a=\frac{4E\sqrt{R}}{3(1-{\upsilon }^{2})}$$

where, R is the radius of the colloidal tip and $\upsilon$ is the Poisson’s ratio. While the modulus obtained by this method does not exactly match that found by bulk testing methods it does offer a significant improvement. One reason for this is the significantly larger indenter geometry, a 4 μm diameter bead compared to an AFM tip with a nominal tip radius of 15 nm. Furthermore the change in shape of the probe is also likely to impact the results obtained and associated wetting and adhesion effects. Using high acuity probes (such a native Berkovich AFM tip) generates high shear and promotes plastic deformation, making them particularly suitable for elastoplastic deformation on materials such as ceramics, while for more viscous samples, such as soft PDMS, small acuity probes, such as spherical probes, are far better suited^36^.

Hence our results do not suggest significant deviations between bulk and local mechanical properties of PDMS materials, especially at higher crosslinker ratios (5% and 10%). We had previously reported that stem cell adhesion and fate decision was remarkably not affected by the mechanical properties of Sylgard PDMS substrates with an extremely wide range of mechanical properties (0.1 kPa to 2 MPa)^6^. This had raised the possibility that local and bulk mechanical properties of PDMS materials significantly differ, although no fundamental basis for such behaviour was proposed^13^. Hence, our results demonstrate that non-biofunctionalised PDMS materials display bulk and nanoscale mechanical properties that are in good agreement. Therefore, the lack of cell response to the bulk mechanical properties of PDMS must have an origin in other phenomena.

It was also previously proposed that the strain rates applied by cells were high enough to result in considerably stiffer perceived moduli, associated with the strong viscoelastic response observed for weakly crosslinked PDMS (1%, Fig. 2a)^13^. Cell sensing of their mechanical environment is proposed to be at velocities in the range of 20–200 nm/s^37^, limited by actin flow rates typically measured in the range of 100–200 nm/s^38^. In comparison, displacements achieved at the periphery of the rheology plate used (where viscoelastic effects should be maximised) are below 2 μm (displacement of the rheometry plate 1 × 10^−4^ rad which translates to 2 μm linear displacement at the edge), indicating that displacements exerted by cells should fall within a frequency range below that used in rheology measurements (<0.1 Hz), a range for which the bulk moduli of PDMS materials still fall well below 1 kPa. Similarly, the indentation velocity used in our AFM measurements was 1 μm/s, a rate that remains significantly higher than those typically applied by cells and that correspond to shear moduli of 30 Pa. Therefore, the viscoelastic profile of weakly crosslinked PDMS materials does not account either for the observed cell spreading on these substrates. Instead, we recently proposed that additives contained in Sylgard 184 PDMS, or modifications introduced during the tethering of extracellular matrix proteins to PDMS materials, are responsible for the increase in nanoscale mechanical properties, at the surface of these substrates, with which cells form adhesions. This behaviour explains, for example, our recent observation of high proliferation of adherent cells (HaCaT epidermal cell line) at the surface of low viscosity liquids, where bulk mechanical properties cannot account for cell adhesion and spreading^39^. In such cases, interfacial rheology measurements gave evidence for the formation of a stiff layer of proteins assembled at the interface between the cell culture medium and the oil used to support cell culture. Similar phenomena could occur at the surface of crosslinked Sylgard 184 PDMS substrates, after biofunctionalisation, therefore changing the nanoscale mechanical properties of the corresponding interfaces.

### Mechanical properties of hydrophilic homogenous PAAm hydrogels

Given the importance of hydrogels for the study of cell adhesion and the development of degradable scaffolds for tissue engineering, multi-scale mechanical characterisation of hydrogels is essential to fully understand interactions between cells and these materials. For such applications, a full understanding of mechanical properties of hydrogels at multiple scales is essential, not only because of their importance to confer structural integrity to the corresponding materials, but also because local deformations and remodelling was shown to have an essential impact on cell phenotype^40,41^. Poly(acrylamide) was used as a model substrate, given its importance for the study of mechanotransduction^6,13,42^. Previous characterisation of PAAm gels via microindentation had indicated that moduli ranged from 2 kPa to 231 kPa, depending on the level of crosslinking and the concentration of monomers used^6,43–46^. However, in the case of PAAm too, a very broad range of moduli are reported for the softest hydrogels (between 1 and 5% crosslinker).

**Figure 5:**
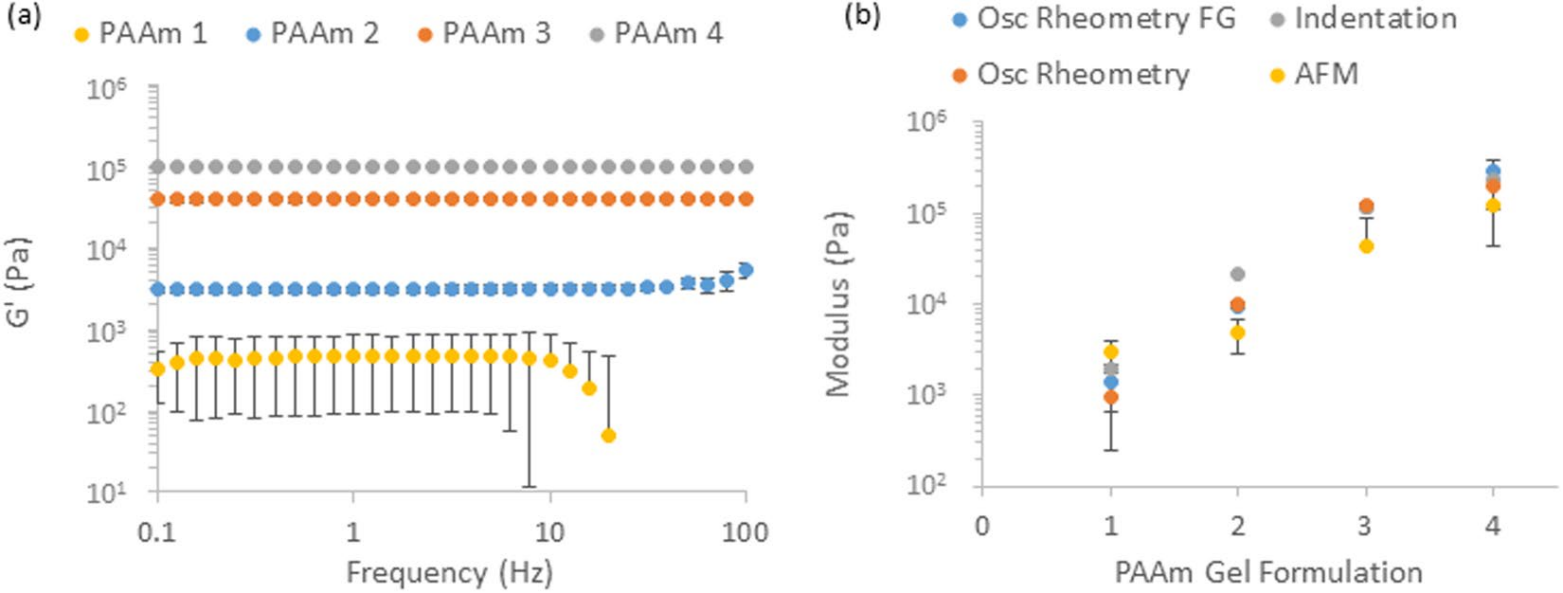
(**a**) Bulk mechanical characterisation of PAAm by oscillatory rheometry (frequency Sweeps) across a range of AAm and crosslinker concentrations (see Table 1 for details of compositions of PAAm1-4, as indicated in the legend), frequencies tested were from 0.1–100 Hz at an oscillating displacement of 10^−4^ rad on a minimum of three repeats and (**b**) comparison of the modulus of the softest PAAm sample measured moduli with and without functionalised geometry and between bulk (rheology and micro indentation) and local (nanoindentation by AFM) testing methods. Error bars showing the standard deviation.

We first investigated further the bulk mechanical properties of this full range of PAAm hydrogels (details of the compositions gathered in Table 1) via rheology (Fig. 5 and Supplementary Fig. S5). Indentation data was in very good agreement with previous results reported, for the full range of compositions tested (Fig. 5b)^6^. In the case of rheology experiments, we generated PAAm hydrogels *in situ* between the two geometries of the rheometer. Results of rheology measurements display the expected general trend of an increase in modulus with increasing crosslinking level and are in good agreement with the microindentation testing previously reported for stiffer samples (1.5% crosslinker and 20% AAm). The moduli of the PAAm samples also showed negligible frequency dependence, apart from the softest samples where there was a significant drop in modulus at frequencies above 10 Hz, this could be explained by fracture of the interface between the geometry and the sample at high frequencies and potentially by the inertia of the testing geometry beginning to dominate the mechanics of the samples for soft PAAm. However at weaker crosslinking levels, the deviation between samples is significantly higher than for the stiffer samples when testing by rheology. Despite this larger spread the data is still in good agreement with the indentation previously measured (moduli of 1400 Pa, compared to 2000 Pa measured before).

We proposed that such discrepancy may originate from two main processes: 1. a lack of adhesion between the plate and the hydrogels generated, resulting in slippage, especially for weakly crosslinked and very hydrated gels; 2. differences in the polymerisation (and gelation) efficiency measured for gels formed *in situ*, compared to those formed for indentation and AFM measurements. The effect of adhesion between the rheometer geometries and the sample was investigated by intercalating two glass coverslips functionalised with methacrylate between the geometries and the samples being crosslinked. Rheological measurements carried out with such functionalised geometries displayed a modest increase in the shear moduli measured at low crosslinker ratio (from 990 Pa to 1400 Pa), indicating that slippage has an impact on shear moduli measured, but that it is not the dominating factor accounting for the discrepancies measured between indentation and rheology. The level of adhesion between the PAAm hydrogel formed and the testing geometries are however clearly enhanced, as was apparent when removing the samples as they clearly detached via cohesive failure, as shown in Supplementary Fig. S6.

**Figure 6:**
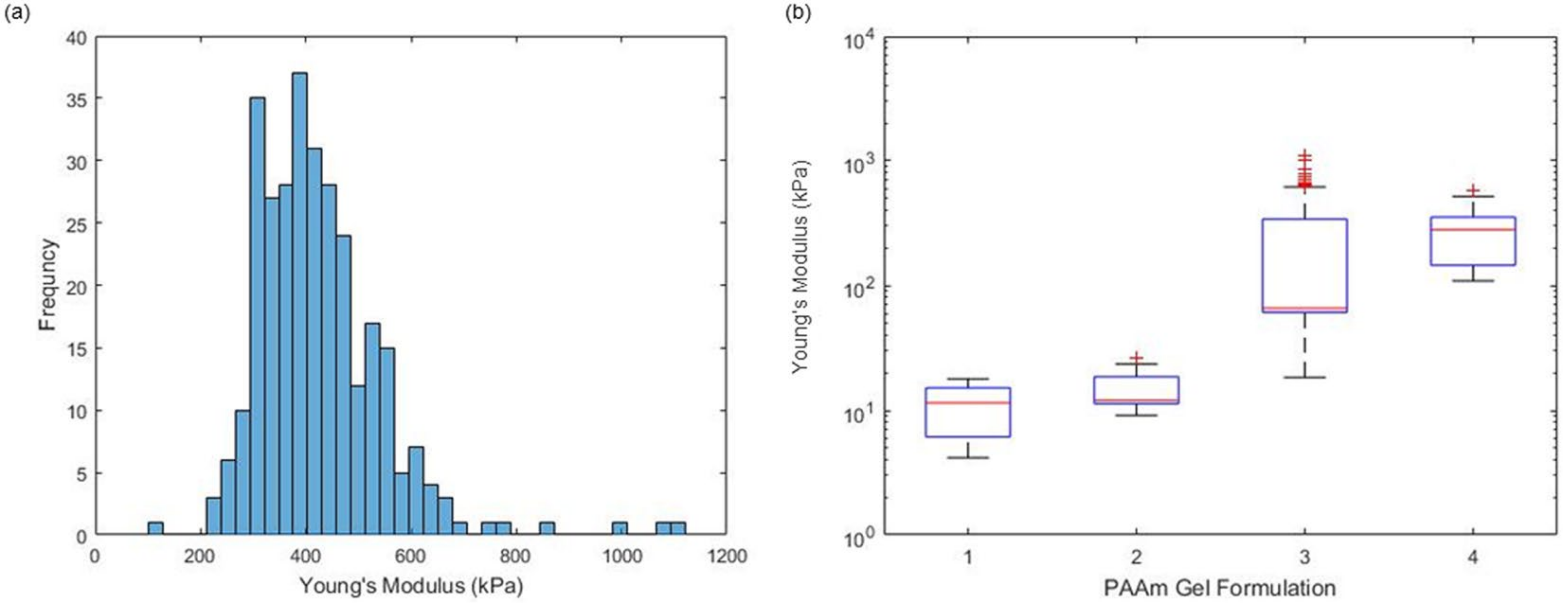
(**a**) Wet AFM data obtained on PAAm sample formulation 3 showing a histogram of the spread of moduli values for 3 repeats of 100 indentations on a 5 × 5 μm sampling area and (**b**) box and whisker plot showing the overall spread across the range of PAAm samples tested. All AFM experiments were conducted with an indentation depth of between 500 and 1000 nm and each curve was done over 1 s, 900 repeats per formulation were conducted with three samples per formulation.

AFM indentation also confirmed the general trend observed between the crosslinker ratios used and the measured elastic modulus of the corresponding materials (Figs 5b and 6). These measurements were in excellent agreement with macroscopic indentation, even in the low range of crosslinking investigated. For each samples tested, the spread of moduli measured was relatively low and gels were found to be relatively homogenous, although we observed a more pronounced variation between samples tested, reflected in the standard deviations. Hence the PAAm hydrogels tested were found to be relatively homogenous, in good agreement with previous results reported for PAAm and PEG methacrylate hydrogels^25^. Indeed heterogeneity and pore sizes for such gels were found to be at a lower scale than that of the cantilever tips used for our studies (nominal tip radius ~20 nm), based on analysis of stress relaxation profiles and the use of poroelasticity models of indentation^6,25^ (pores below 20 nm). Therefore heterogeneity is unlikely to be the origin of the discrepancies between rheology and indentation data. In addition, very little adhesion was observed in AFM indentation traces at all PAAm moduli (Supplementary Fig. S7). This is perhaps expected as PAAm is only very weakly adhesive and a neutral gel resulting in little sample-tip interaction. Hence discrepancies between rheology and indentation testing is most likely explained by the different methods of sample preparation where the quality of the gel network is heavily influenced by any contact with oxygen during gelation.

### Mechanical properties of crosslinked carboxymethyl cellulose hydrogels

**Figure 7:**
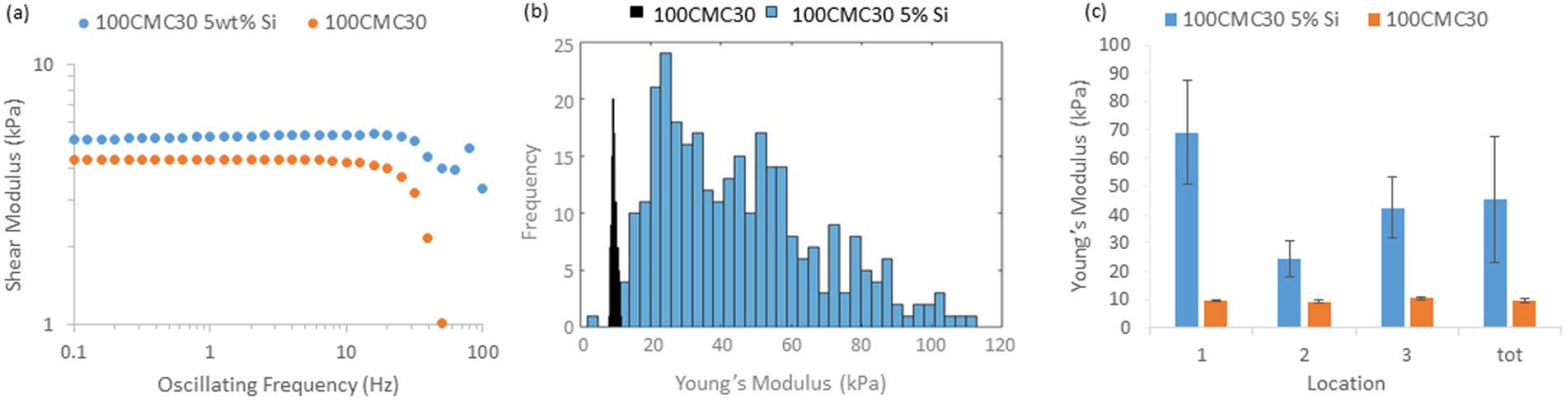
(**a**) Representative oscillatory frequency sweeps on 100CMC30 with and without Si beads, frequencies of oscillation were from 0.1–100 Hz at an oscillating displacement of 10^−4^ rad showing no frequency dependence (at the highest frequencies the inertia of the testing geometry dominates over the mechanics of the gel resulting in the decrease in measured modulus) and (**b**) Histogram comparing the moduli of 100 CMC30 with and without Si beads, the large spread of data with Si beads illustrating the impact heterogeneity has on local mechanical testing. All AFM experiments were conducted with an indentation depth of between 500 and 1000 nm and each curve was done over 1 s. (**c**) Summary of AFM tests performed on 100CMC30 with and without Si beads highlighting the difference resulting from heterogeneity on mechanical testing with a black line representing the moduli of CMC samples obtained by oscillatory rheology.

In contrast to poly(acrylamide) hydrogels that do not show significant levels of heterogeneity and do not lead to significant adhesion with the surfaces of probing geometries, other charged hydrogels and composite structures are often associated with significant discrepancies between local and bulk moduli^25,29^. We used a carboxymethyl cellulose (CMC) hydrogel as a case study, for comparison to PAAm hydrogels previously discussed. Since CMC is not covalently crosslinked, we introduced additional crosslinks via allyl pendant chains that react with poly(ethylene) glycol dithiol crosslinkers via thiol-ene coupling^47^. This type of reactions was of particular interest because of its relevance for *in situ* cell encapsulation within 3D hydrogels^48^, under mild conditions and its good tolerance of moderate oxygen concentrations^49,50^. Two allyl CMC hydrogel samples were tested, both at 100 mg/mL CMC concentrations, with and without the addition of 5 wt% silica beads (displaying a composite morphology with local heterogeneity). The two samples were characterised via oscillatory rheology and nanoindentation (Fig. 7 and Supplementary Fig. S8).

Oscillatory rheology displayed classical frequency independent elastic profiles (up to 30–40 Hz) for both CMC samples, with and without silica beads, with very similar bulk moduli (16.5 and 13.5 kPa, respectively). Above 40 Hz we see a significant drop in the shear storage modulus, this could be explained by the fracture of the interface between the sample and the geometry and the inertia of the testing geometry dominating the mechanics of the sample itself, as stated for the softest PAAm, however as these samples are significantly stiffer than the softest PAAm, and considering the high molecular weight of the CMC molecules used in the preparation of these gels, it is also possible that some shear thinning occurs.

Despite the rheology data on the two CMC gels being in good agreement, when characterised by AFM indentation, they displayed significantly different behaviours as shown in Fig. 7. The CMC gel incorporating silica beads displayed a significantly higher modulus (45 kPa) compared to that of the same gel without any added beads (10 kPa). In addition, the spread in the modulus within one sample was significantly broader for the composite hydrogel, clearly indicating a high level of heterogeneity (Fig. 7). Therefore, rheology and AFM data are in excellent agreement for the CMC gel investigated, but display significant discrepancy for the composite hydrogel. This is thought to arise at low loading levels of fillers, for which a percolated network has not been achieved yet, hence resulting in a negligible impact on bulk mechanical properties whilst the local modulus of the sample at or close to silica beads will appear significantly higher than that of the surrounding soft material.

## Conclusions

When comparing the mechanical characterisation data obtained across the three systems studied, we clearly observe very good agreement between bulk and local mechanical testing methods for the stiffest samples. However this agreement breaks down when testing soft materials (moduli of the order of kPa), in particular for the soft PDMS samples. Our results clearly identify potential origins for the discrepancy between local and bulk mechanics. Wetting and adhesion cause fouling of the local testing geometry when performing nanoindentation. This results in an increase of the contact area and therefore a decrease of the true stress applied to the sample. We propose that this phenomenon explains the apparently stiffer modulus measured for 1% crosslinked Sylgard 184 PDMS when tested by AFM indentation. Despite reducing the amount of adhesion between the AFM tip and the PDMS sample by performing the tests in ethanol solution, adhesion between the geometry and the sample could not be totally eliminated. This phenomenon was not as prevalent in the case of hydrogels such as PAAm, owing to the hydrophilicity and neutral charge of these materials. It is therefore important to consider the tip sample interaction when deciding the best method for mechanical testing.

Heterogeneity was also found to have a significant effect on the mechanical properties obtained when using local testing methods. This phenomenon was highlighted within the allyl CMC loaded with silica nanoparticles, where local stiffening was observed by AFM. However the dimensions of phases required to have an effect on the measured modulus should be comparable to the probe size. This contrast with the impact of heterogeneity on viscoelastic and poroelastic profiles, which can be sensed by AFM indentation even in the case of features orders of magnitude smaller than the probe diameter^51^.

In conclusion, the characterisation of soft materials at multiple scale can be influenced by a number of factors that should be examined in order to validate the methodology used for such comparisons. Adhesion and heterogeneity in particular, alongside potential differences between sample preparations, have been highlighted as possible causes of discrepancies between bulk and local mechanical testing. These phenomena are, however, not universal and the characterisation of soft biomaterials should involve the analysis of the impact of such factors to validate the methodology selected.

## Electronic supplementary material

Supplementary Information
