# Supplementary material for: Impact of surface adhesion and sample heterogeneity on the multiscale mechanical characterisation of soft biomaterials
Source: Sci Rep. 2018 Apr 30;8:6780. doi: 10.1038/s41598-018-24671-x (PMC5928067; doi:10.1038/s41598-018-24671-x)
Supplement: Supplementary file 1 — Supplementary Information [file 41598_2018_24671_MOESM1_ESM.pdf]

Impact of surface adhesion and sample heterogeneity on the multiscale mechanical characterisation of soft biomaterials

W. Megone[a, b], N. Roohpour[c] and J. E. Gautrot[a, b] *

[a]Queen Mary University of London, Mile End Road, London E1 4NS, UK

[b]Institute of Bioengineering, Queen Mary, University of London, Mile End Road, London, E1 4NS, UK.

[c]Consumer Healthcare R&D, GlaxoSmithKline, St George's Avenue, Weybridge, Surrey, KT13 ODE, UK.

* To whom correspondence should be addressed, e-mail: j.gautrot@qmul.ac.uk.

Supplementary Information.

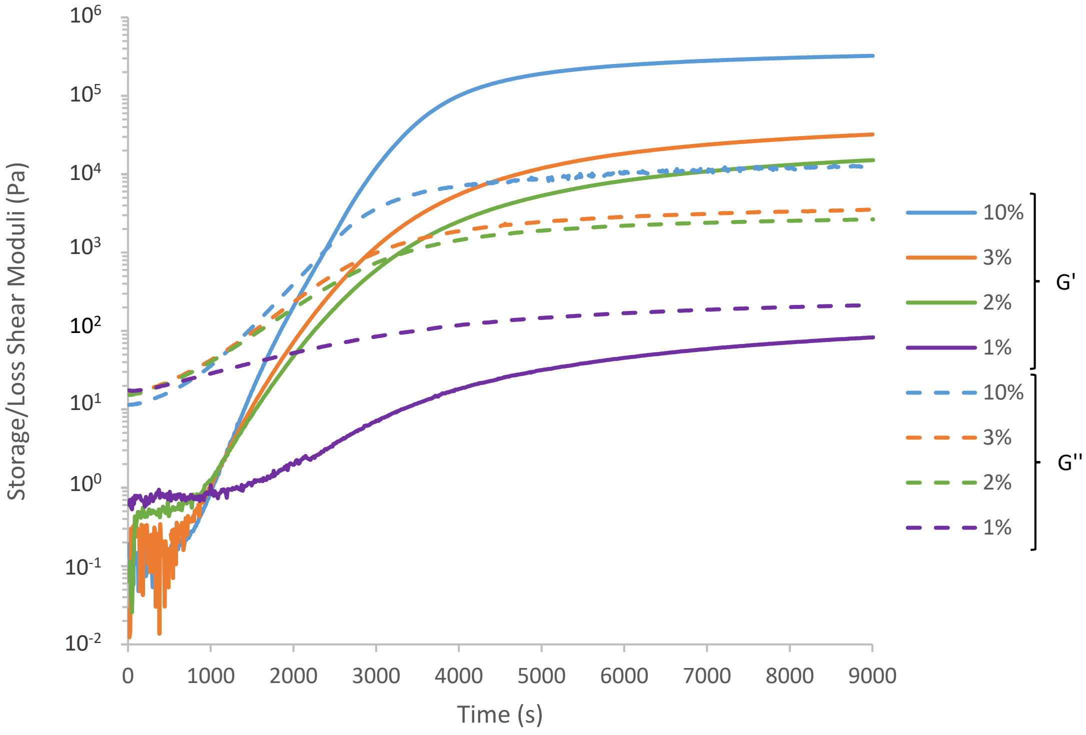

Figure S1. Oscillatory rheology time sweeps monitoring the curing of PDMS at different base-crosslinker concentrations (indicated in the legend, the % referring to the weight % of crosslinker relative to the base). Time sweeps were conducted at a frequency of 1Hz and with a displacement of 10^{-4} rad.

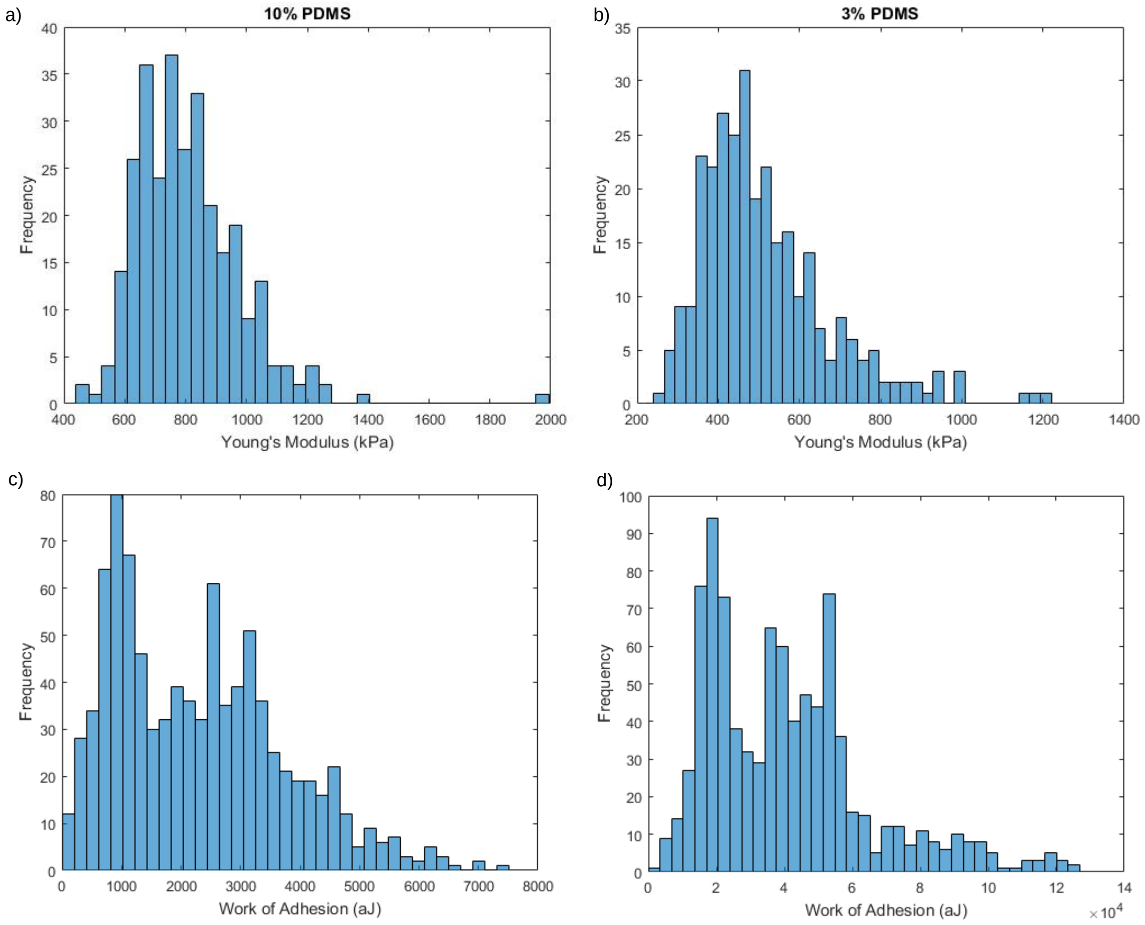

Figure S2. Histograms showing the distributions in Young's moduli obtained by AFM on PDMS samples tested dry for PDMS samples with (a) 10% crosslinker by weight and (b) 3%. Each sample is tested in three 5X5um locations with 100 indentation tests spread evenly across each location and the spread in the work of adhesion for PDMS with (a) 10% and (b) 30% crosslinker

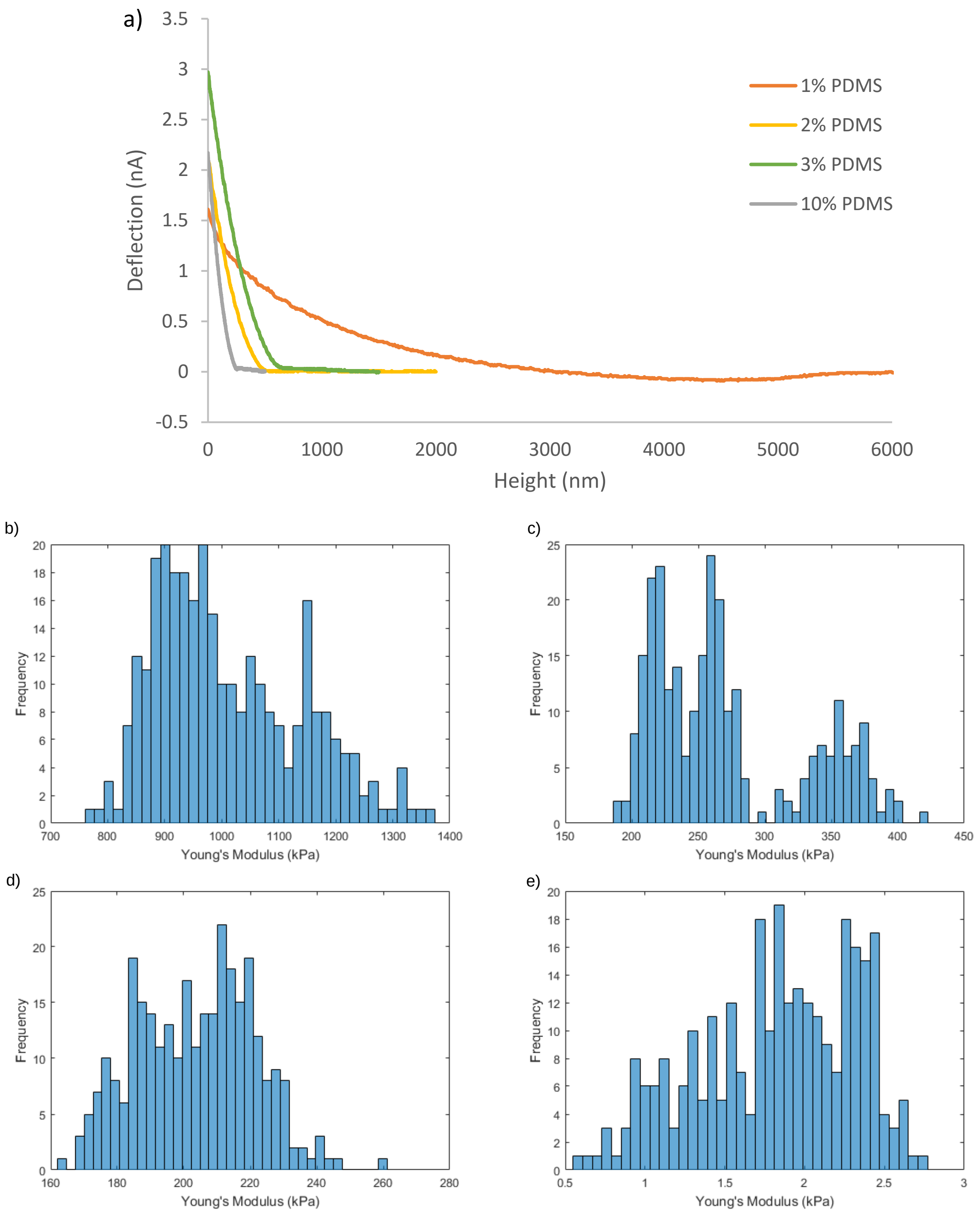

Figure S3. (a) Raw AFM retraction curves for PDMS at varying crosslinker concentrations tested in ethanol solution with a 4 μm bead attached to the AFM tip. The indentation depth was around 500 nm and each curve was done over 1s. For the softest sample adhesion is still observed. (b), (c), (d) and (e) histograms of the Young's moduli obtained for the PDMS samples with 1%, 2%, 3% and 10% crosslinker respectively. Each sample is tested in three 5 by 5 μm locations with 100 indentation tests spread evenly across each location.

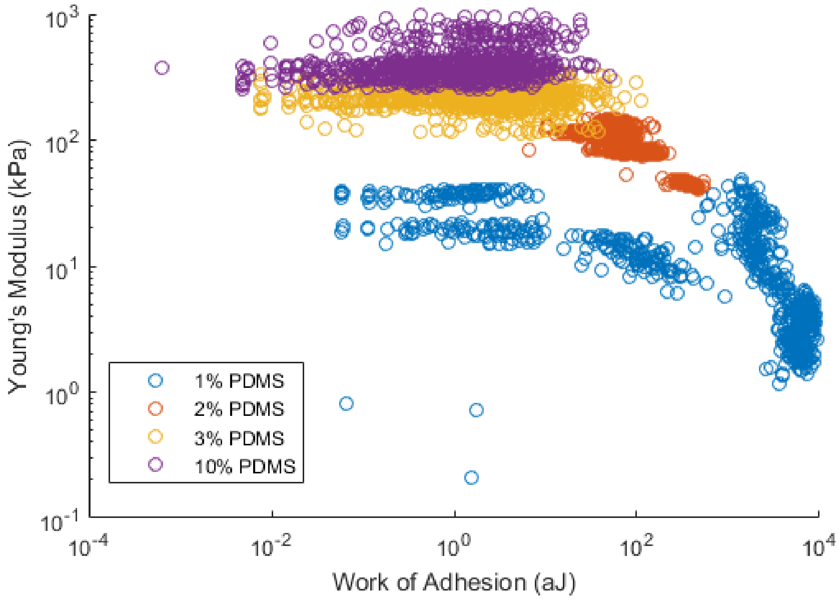

Figure S4. AFM data correlating the Young's modulus on PDMS samples with 1 – 10% crosslinker as a function of the work of adhesion. Three repeats on three samples per formulation giving 900 points per sample. Each individual sample is tested in three 5 by 5 μm locations with 100 indentation tests spread evenly across each location.

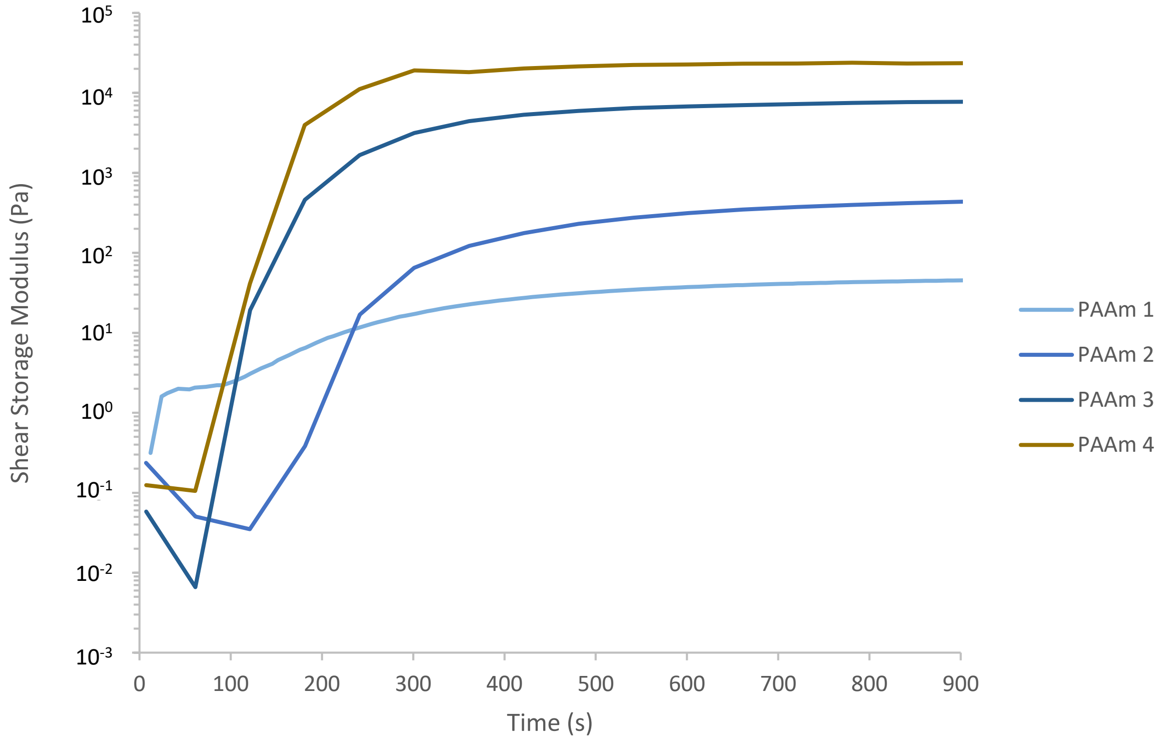

Figure S5. Oscillatory rheology time sweeps monitoring the curing of PAAm gels with different compositions. The legend indicates which trace corresponds to gels 1-4 (see Table 1). Note that gelation of samples for analysis was allowed to continue without oscillation for 1.5 h time. Time sweeps were conducted at a frequency of 1Hz and with a displacement of 10^{-4} rad.

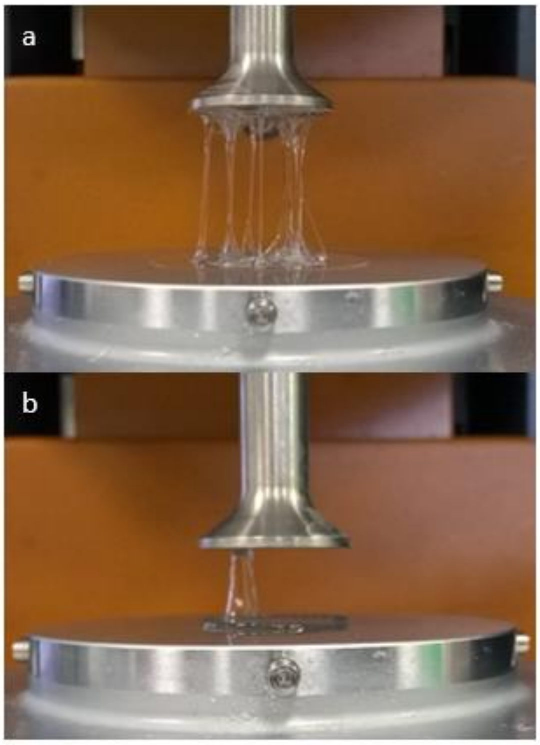

Figure S6. Adhesion between PAAm samples (gel 1) to the rheometer geometry when coming out of contact with (a) and without (b) methacrylate-functionalised geometries.

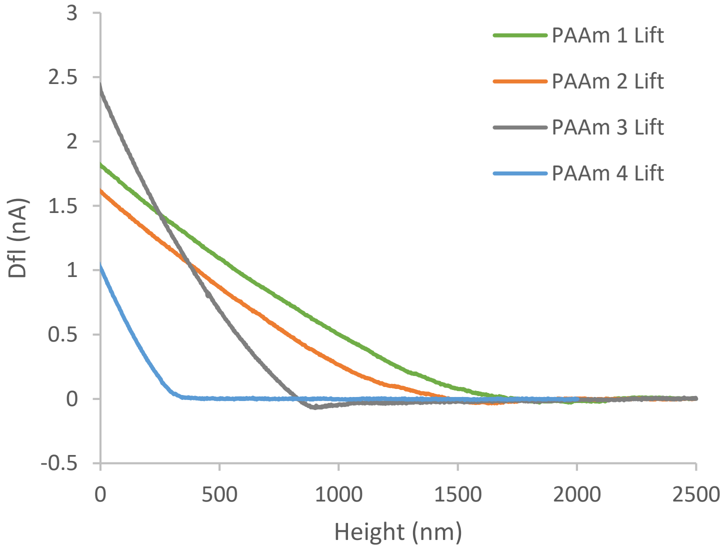

Figure S7. Raw AFM retraction curves for the series of PAAm samples tested (see Table 1) with a 4 μm bead attached to the AFM tip. The indentation depth was between 300 and 1000 nm was and the curves were completed over 1s.

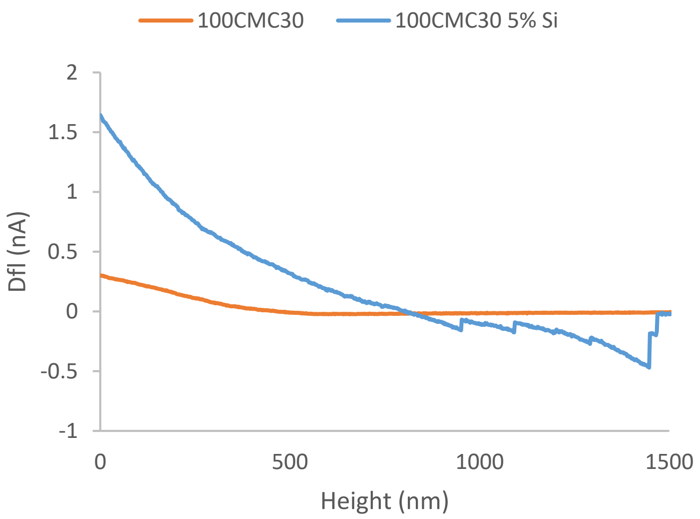

Figure S8. Representative AFM lift curves for CMC gels with and without Si beads mixed into the sample. The indentation depth were kept between 500 and 1000 nm and each indentation was carried out over 1s.
